# Supplementary material for: Malaria parasites regulate intra-erythrocytic development duration via serpentine receptor 10 to coordinate with host rhythms
Source: Nat Commun. 2020 Jun 2;11:2763. doi: 10.1038/s41467-020-16593-y (PMC7265539; doi:10.1038/s41467-020-16593-y)
Supplement: Supplementary file 1 — Supplementary Information [file 41467_2020_16593_MOESM1_ESM.pdf]

This document includes:

Supplementary Fig. 1 to 7
Supplementary Table 1

Supplementary Information

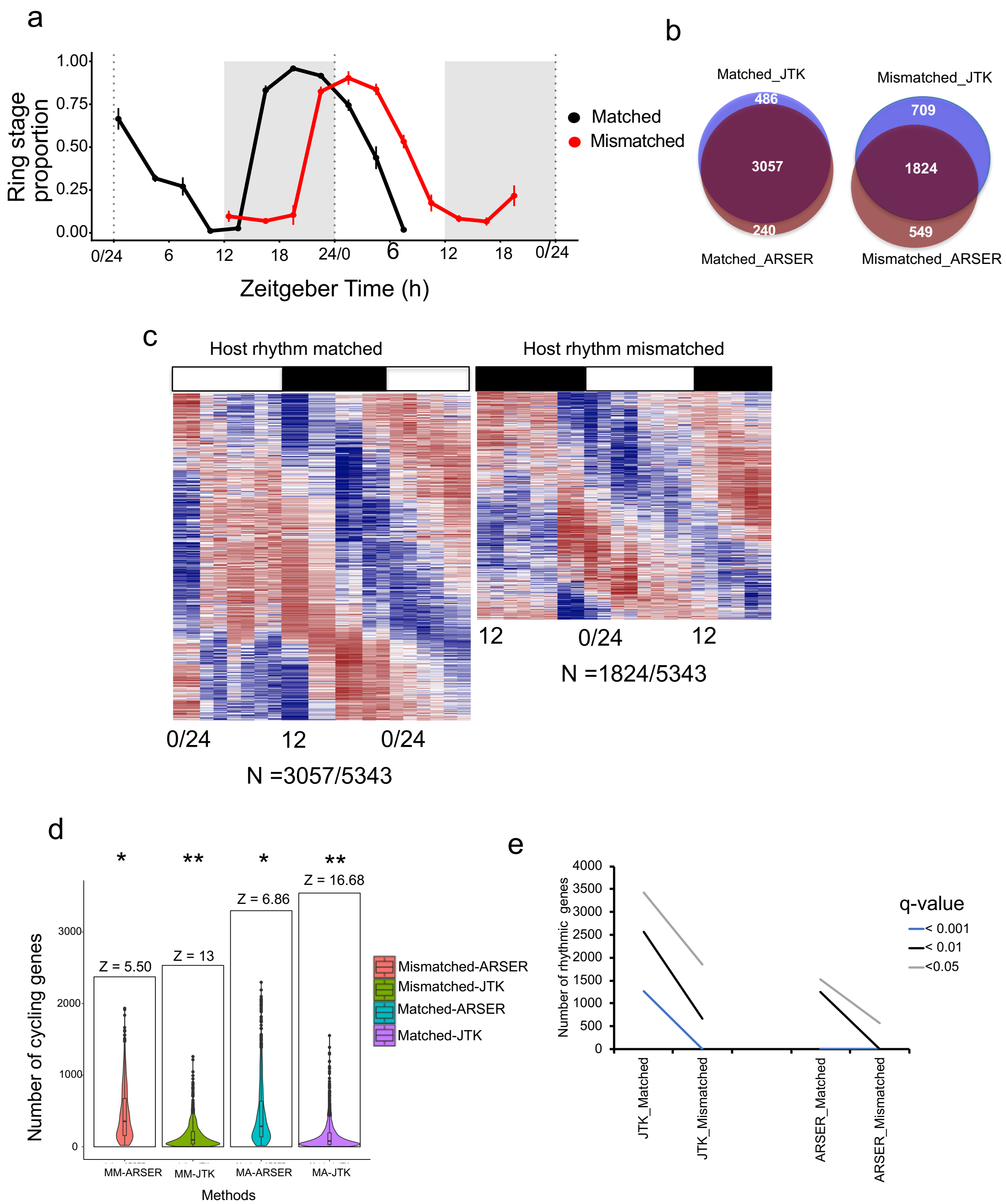

Supplementary Figure 1| Rhythmic expressed genes in host rhythm matched and mismatched parasites

a) Ring stage proportion of matched (black line) and mismatched (red line) parasites across 11 time points (Mean ± SEM, N = 4 per time point).

b) Venn diagram of the number of daily rhythmic transcripts ($p< 0.05$, see Methods) identified by two algorithms in matched and mismatched parasites. All the daily rhythmic transcripts used in this analysis are listed in Supplementary Data 1.

c) Time series gene expression heatmap views of transcripts with daily rhythmicity in matched (heatmap on left) and mismatched (heatmap on right) parasites. Each row in heatmap represents a single gene, sorted according to the phase of maximum expression starting from first sample time point. The phase of expression of each gene was obtained from ARSER output and N represents number of genes identified by both JTK and ARSER as fluctuating in expression in a 24 h manner. Each time point is represented by expression heatmap of two biological replicates.

d) Number of daily rhythmic genes identified by two algorithms employed in the correct order of sampling time points (black bars). Violin plot represents the distribution of daily rhythmic genes identified by permuting the order of sampling time points 1000 times.

For boxplots inside the violin plots, centerline indicates the median, box limits indicate upper and lower quartiles, whiskers indicate the 1.5 interquartile range and points indicate outliers and * represents statistical significance between the number of daily rhythmic genes between in real order and permuted order at FDR < 0.01, ** represents FDR < 0.001. Z represents the Z score.
e) Number of daily rhythmic genes identified at different ranges of q values by ARSER and JTK in host-rhythm matched and mismatched parasites. Source data are provided as a Source Data file.

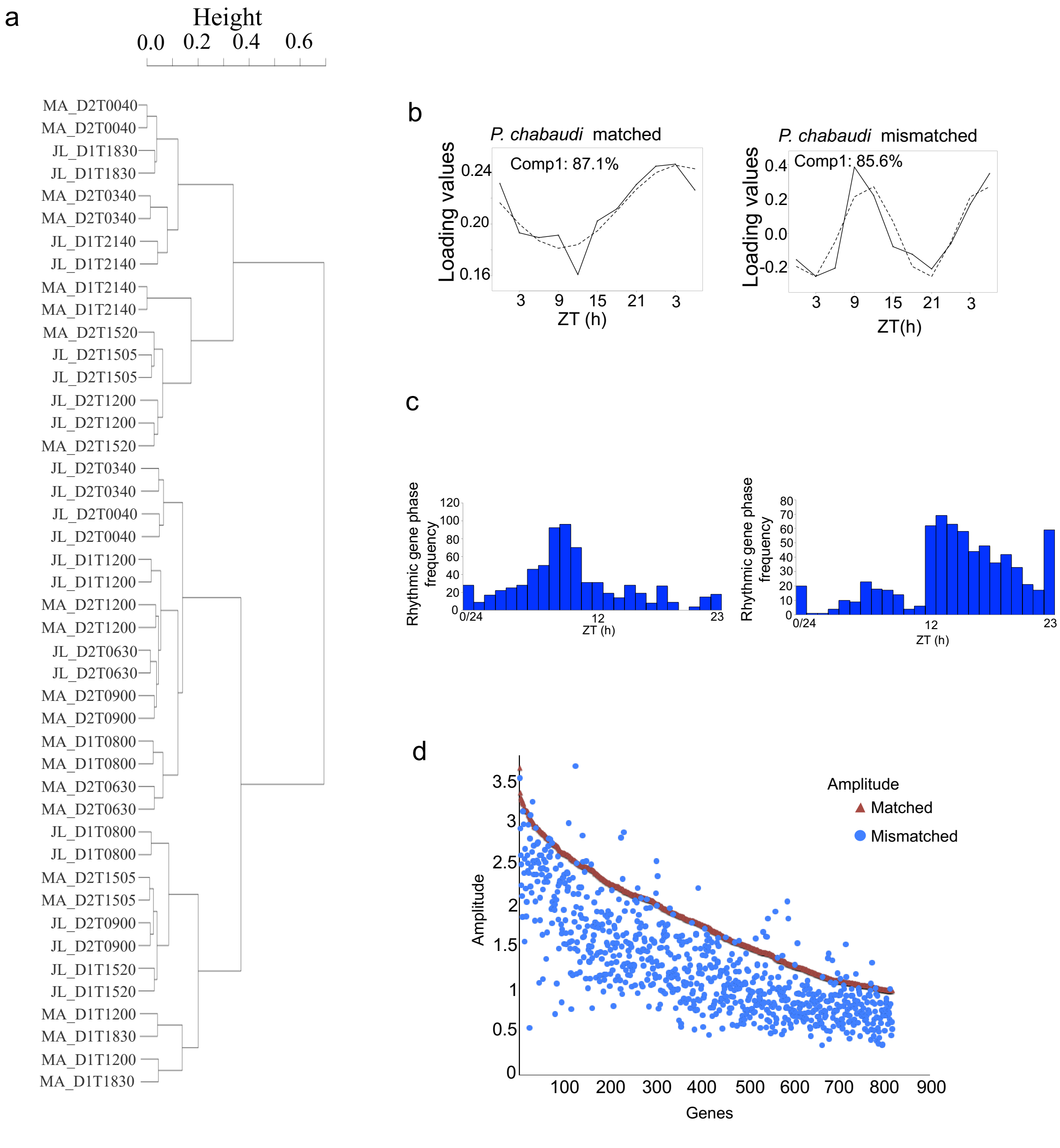

Supplementary Figure 2 | Mismatching affects the phase of expression and amplitude of rhythmically expressed genes.

a) Clustered dendrogram of 11 time points with two biological replicates per time points using hierarchical clustering algorithm. Analysis was performed on normalized count data Abbreviations: MA, matched; JL, mismatched
b) Principal component analysis (PCA) from matched and mismatched parasites representing cyclic component in solid line and dashed line of its best-fitted cosine curve, which is the first component of the PCA.
c) Bar chart represents the phase of expression of 685 daily rhythmic genes in matched and mismatched parasite that had delayed phase of ~6 h in matched compared to mismatched parasites. Each bar represents the number of rhythmic genes in that phase of the da
d) Scatter plot between amplitude of 817 daily rhythmic genes from host rhythm matched and mismatched parasites with high time series amplitude (> 1) in host rhythm matched parasites. Genes in host rhythm mismatched parasites are sorted in descending order based on amplitude. Source data are provided as a Source Data file.

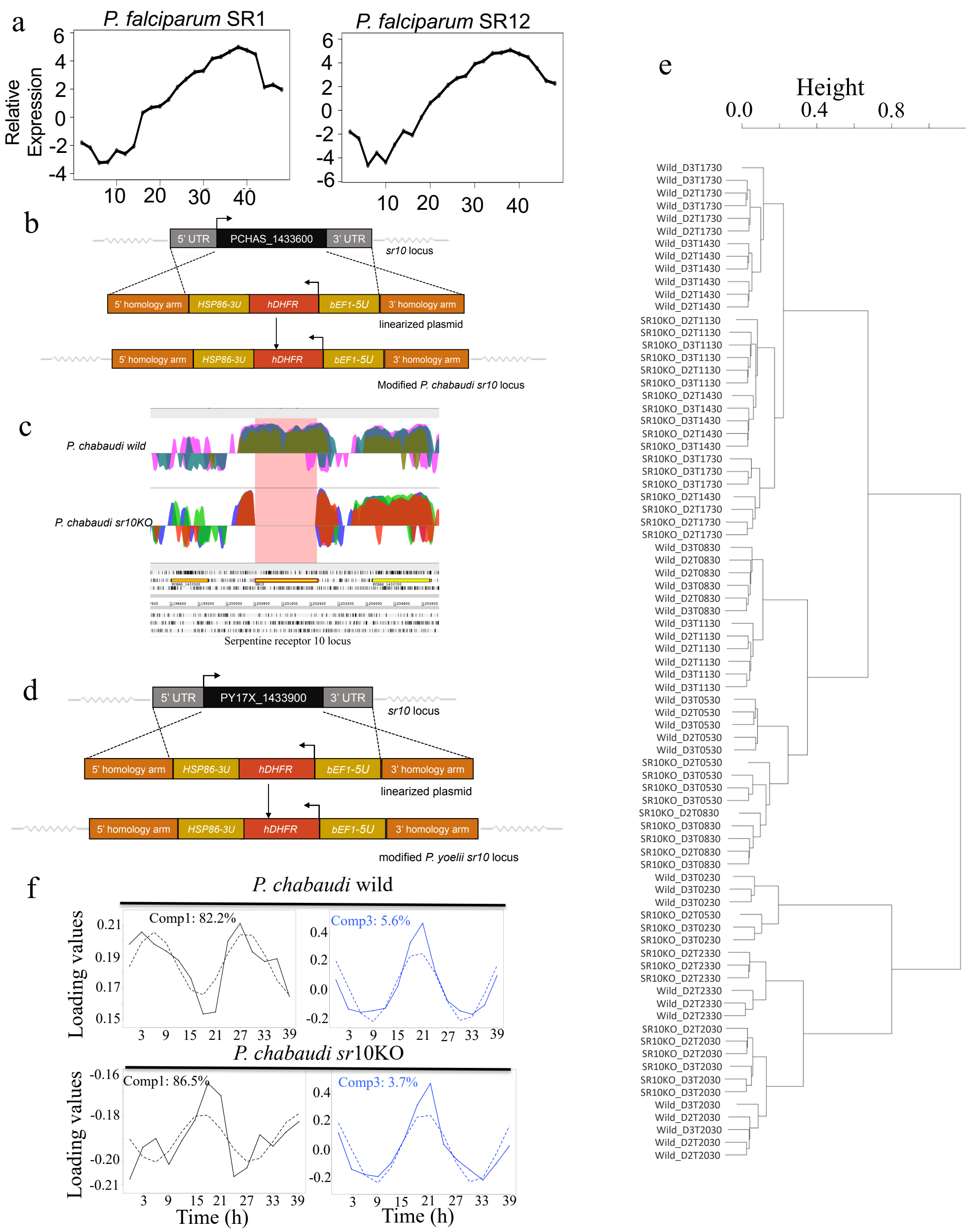

Supplementary Figure 3| Confirmation of *sr10* knockout

a) Expression profiles of *P. falciparum* SR01 and SR12 over 48 h intra-erythrocytic developmental cycle.

b) Schematic of *sr10* knockout strategy in *P. chabaudi*. Linearized plasmid contains a human dihydrofolate reductase-thymidylate synthase (hDHFR-TS) under the control of *P. berghei* EF1 alpha promoter and *P. falciparum* HSP86 3' UTR terminator which is flanked by 1000 bp long regions homologous to 5'UTR and 3'UTR of PCHAS_1433600. Upon integration, the complete PCHAS_1433600 coding region is replaced with the hDHFR drug resistance cassette.

c) Confirmation of *sr10* knockout in *P.chabaudi* using RNAseq. Artemis view of *sr10* locus in *P. chabaudi* wild and *P. chabaudi sr10*KO parasites. *sr10* locus is highlighted. As seen clearly, reads from wild parasites mapped perfectly in the *sr10* region where as no reads from *sr10*KO parasites mapped to the same genomic region. Sequencing reads from 4 randomly chosen time-points from

each parasite strains are shown. PCR and Sanger sequencing based evidences supporting successful knockout of *sr10* in *P. chabaudi sr10*KO and *P. yoelii sr10*KO are provided in Supplementary Fig. 7 and Supplementary Data 9

d) Schematic of *sr10* knockout strategy in *P. yoelii*. Linearized plasmid contains a human dihydrofolate reductase-thymidylate synthase (hDHFR-TS) under the control of *P. berghei* EF1 alpha promoter and *P. falciparum* HSP86 3' UTR terminator which is flanked by 1000 bp long regions homologous to 5'UTR and 3'UTR of PY17X_1433900. Upon integration, the complete PY17X_1433900 coding region is replaced with the hDHFR drug resistance cassette.

e) Clustered dendrogram of 14 time points with two biological replicates per time points using hierarchical clustering algorithm. Analysis was performed on normalized count data.

f) Principal component analysis from wild type and *sr10*KO parasites representing cyclic component in solid line and best fitted cosine curve in dashed line which are first and third components of the PCA.

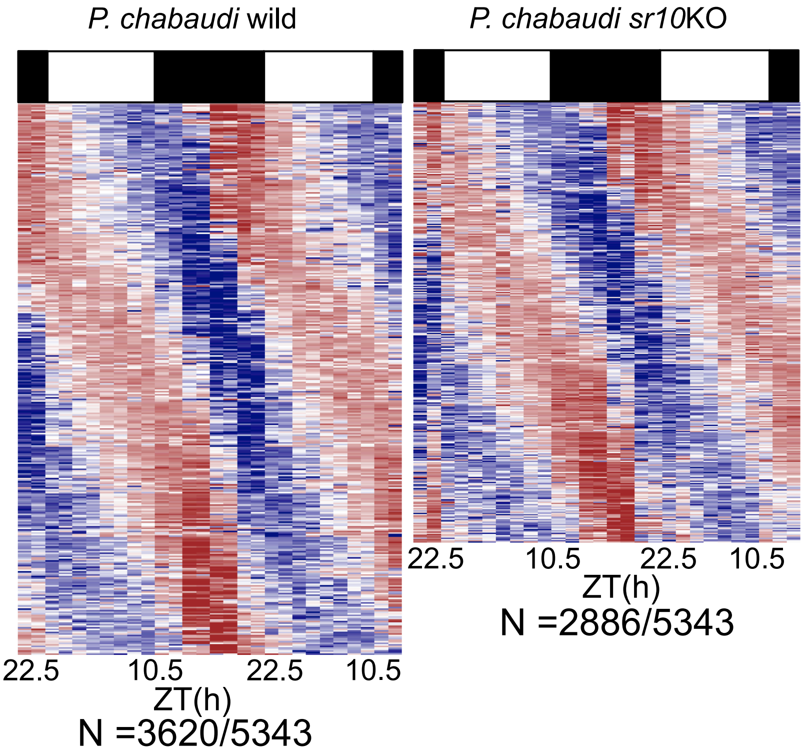

Supplementary Figure 4 | Daily rhythmic transcripts in wild and *sr10*KO parasites

Time series gene expression heatmap views of transcripts with daily rhythmicity in wild type (heatmap on left) and *sr10*KO (heatmap on right) parasites. Each row in heatmap represents a single gene, sorted according to the phase of maximum expression starting from first sample time point. The phase of expression of each gene was obtained from ARSER output and N represents number of genes identified by both JTK and ARSER as fluctuating in expression in a 24 h manner. Each time point is represented by expression heatmap of two biological replicates.

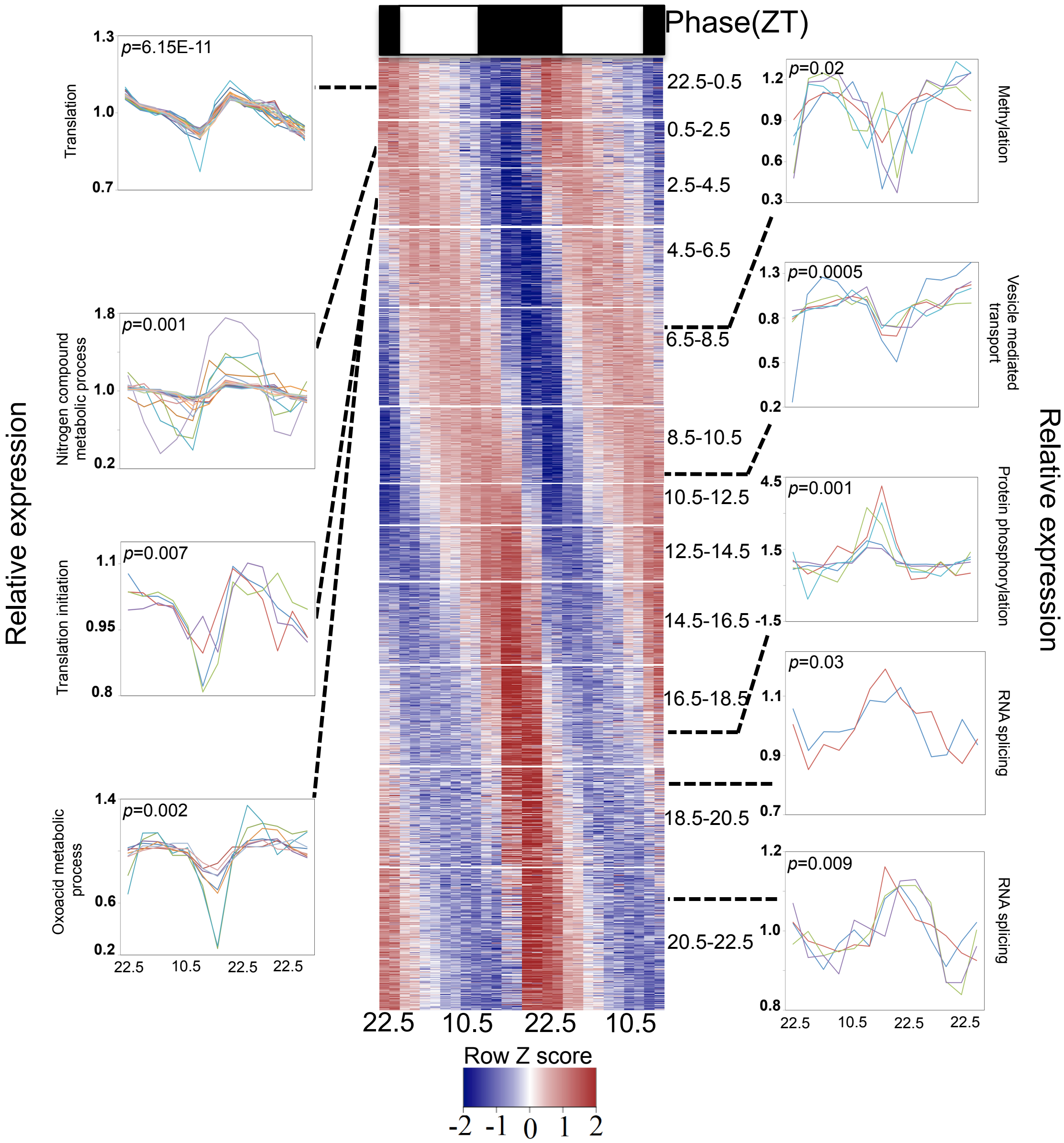

Supplementary Figure 5 | Disruption of *sr10* affected circadian genes associated with multiple metabolic processes
Time series gene expression view of daily rhythmic genes that lost rhythmicity in mismatched parasites. The heat map has been segregated into 12 parts with each part representing 2 h phase clusters. Genes were sorted based of phase of expression. Line plots along the sides of the heat map represent expression profiles of individual genes from the significantly enriched gene ontology terms (false discovery rate corrected $p<0.05$, hypergeometric test, one-sided) in few selected phase clusters. Each plot has information about the false discovery rate corrected p value of representing gene ontology term. Y axis represents relative expression of genes in each time points which was determined by expression count of each genes normalized by its mean derived from 14 time points.

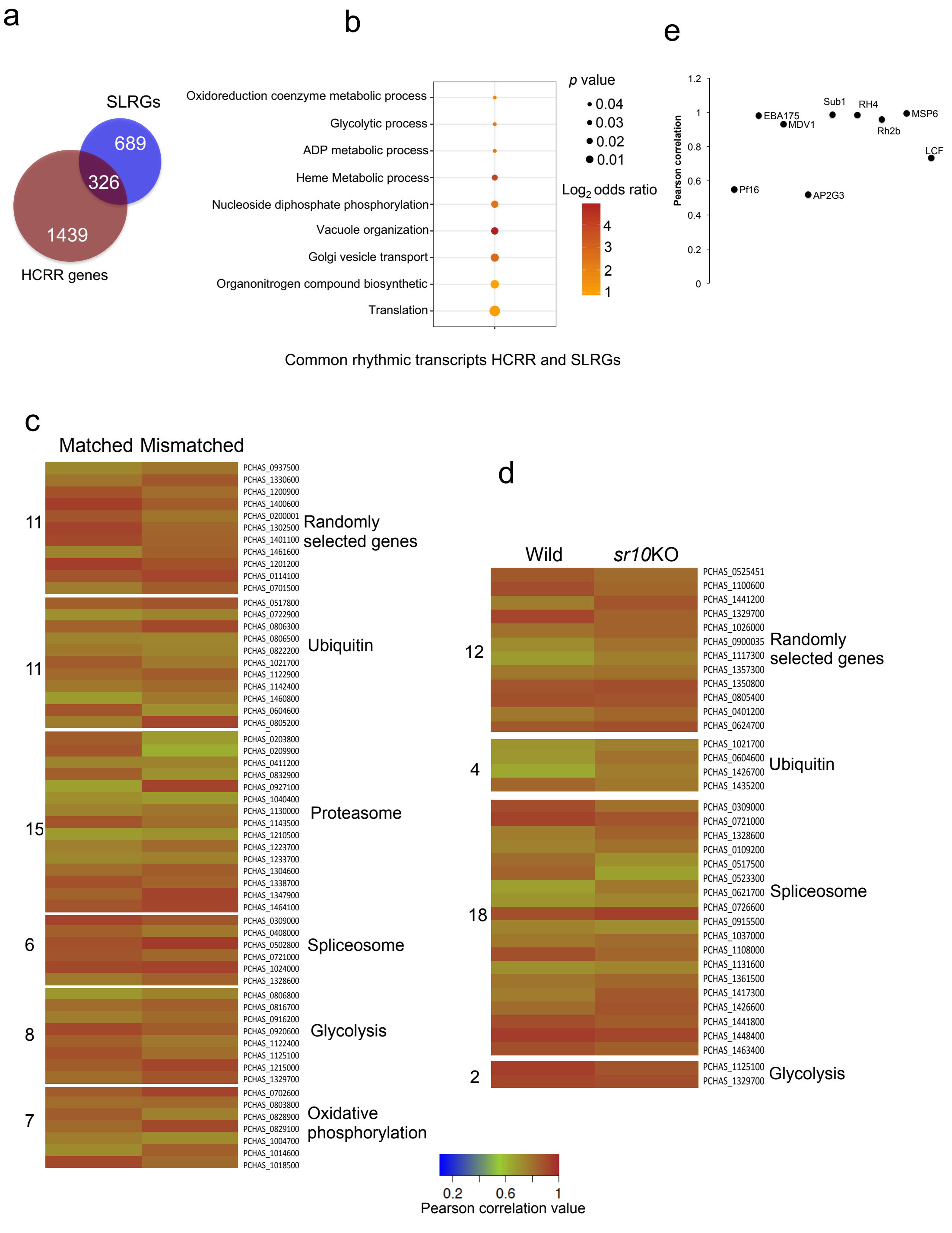

Supplementary Figure 6 | Comparison of daily rhythmic genes with dampened rhythmicity in mismatched parasites and *sr10*KO parasites.

a) Venn diagram comparing host cues responsive rhythmic genes (HCRR genes) and *sr10* linked rhythmic genes (SLRGs). Daily rhythmic genes in matched parasites that lost daily rhythms in host-rhythm mismatched parasites (HCRC genes) were compared with daily rhythmic genes that lost their rhythm in *sr10*KO parasites compared to wild-type parasites (SLRGs).

b) Gene ontology enrichment analysis of genes common between host cues responsive circadian genes and SR10 linked circadian genes. Manually curated enriched gene ontology terms (false discovery rate corrected $p<0.05$, hypergeometric test, one-sided) have been represented.

c) Heat map representing correlation values between high-throughput qPCR (HT qPCR) and RNASeq data for matched and mismatched parasites. A total of 58 genes were validated.

d) Heat map representing correlation values between HT qPCR and RNASeq data for *P. chabaudi* wild and *P. chabaudi sr10*KO parasites. A total of 36 genes were validated.
RNASeq expression data from 8 time points were compared with HT qPCR expression data from 8 time points to obtain the Pearson correlation values. For RNASeq, normalized count values were used, whereas for qPCR, transcript values (Ct) of genes were normalized to non-cycling transcripts of U5 small nuclear ribonucleoprotein component, putative (PCHAS_1202900). Two technical replicates and two biological replicates were used per time points for HT qPCR experiment. Two biological replicates were used per time points for RNASeq experiment.
e) Dot plot showing the Pearson correlation values between qPCR and RNASeq data from *P. falciparum* time-series experiment. Source data are provided as a Source Data file.

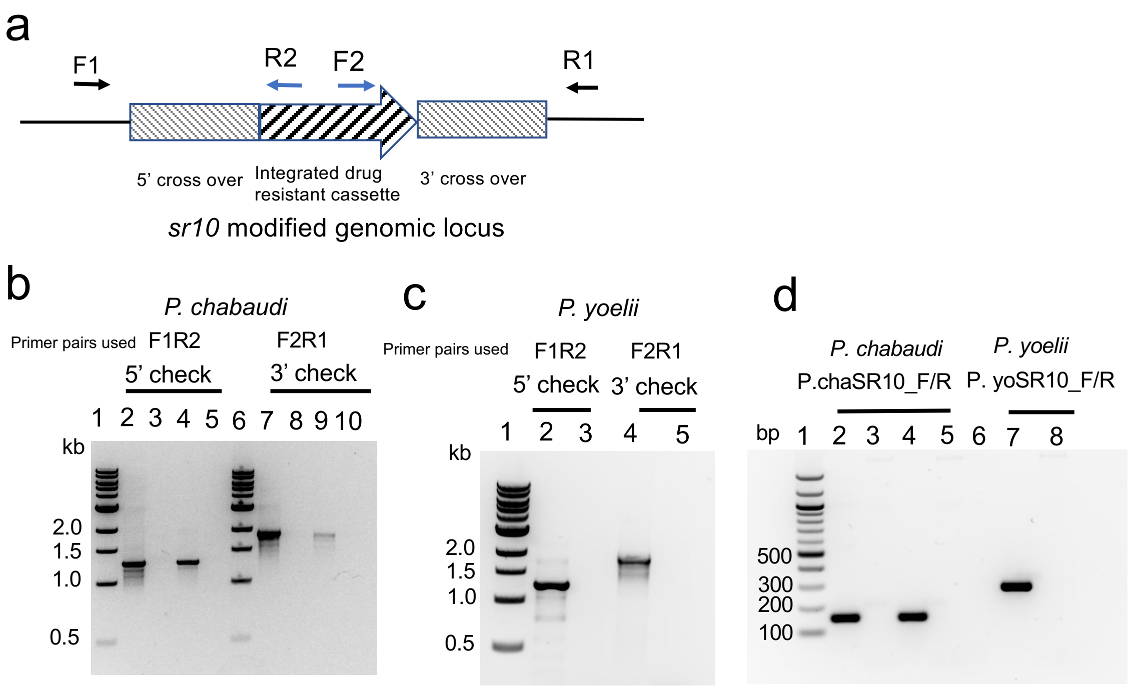

Supplementary Figure 7| Confirmation of *sr1*0 knockout in *P. chabaudi* and *P. yoelii*
a) Schematic of gene locus after *sr10* knockout with integration of drug resistant cassette. Arrow mark shows primers used to confirm successful knockout of *sr10* in *P. chabaudi* and *P. yoelii*. Conserved sequence between *P. chabaudi* and *P. yoelii* were used to design external forward (F1) and reverse (R1) primers. Primers internal to drug resistant cassette (R2 and F2) used in combination with external primers to confirm *sr10* knock out. Amplification from primer pair F1R2 will confirm 5' integration and amplification from primer pair F2R1 will confirm 3' integration of drug resistant cassette in the *sr10* locus.
b) PCR screen on *P. chabaudi sr10* KO A and *P. chabaudi sr10* KO B clones confirming the successful integration of drug resistant cassette replacing the wild *sr10*. Lane 1, 1kb ladder, Lane 2-5, *P.chabaudi sr10* KOA, *P.chabaudi* wild-type, KOB and *P.chabaudi* wild-type clones respectively; Lane 6, 1kb ladder, Lane 7-10, *P.chabaudi sr10* KOA, *P.chabaudi* wild-type, KOB and *P.chabaudi* wild-type clones respectively.
c) PCR screen on *P. yoelii sr10* KO clone confirming the successful integration of drug resistant cassette replacing the wild *sr10* Lane 1, 1kb ladder, Lane 2-5, *P. yoelii sr10* KO, wild-type, *P. yoelii sr10* KO, and wild-type clones respectively.
d) PCR screen on *P. chabaudi sr10* KO A, *P. chabaudi sr10* KO B and *P. yoelii sr10* KO confirming the absent of wild *sr10.* Primer pair P.chaSR10_F and R was used to detect P. *chabaudi sr10* and primer pair P.yoSR10 F and R was used to detect *P. yoelii sr10*. In all the sr10KO clones sr10 was not detected while sr10 was detected in wild *P. chabaudi* and *P. yoelii* parasites. Lane 1, 100 bp ladder, Lane 2 and 4, *P. chabaudi* wild, Lane 7, *P. yoelii* wild, Lane 3 and 5, *P. chabaudi sr10*KOA and *sr10*KOB and Lane 8, *P. yoelii sr10*KO, Lane 6, blank.
Experiments in panels b,c and d were repeated twice, with reproducible results.

Supplementary Table 1: Rhythmicity characteristics of wild type and *sr10*KO parasites.

Parasite Strain	Parasite Stage	n	Amplitude (mean ± se)	Period (mean ±se)	Phase (mean ±sd)	Mean CoG (mean ±sd)
Pch *sr10*KO A	early_trophs	4	0.79 ± 0.02	22.35 ± 0.43	3.19 ± 0.34	0.71 ± 0.07
Pch *sr10*KO B	early_trophs	4	0.93 ± 0.03	22.45 ± 0.24	1.63 ± 0.16	23.45 ± 0.03
Pch WT	early_trophs	4	0.94 ± 0.03	25.15 ± 0.37	0.16 ± 0.25	2.04 ± 0.04
Pyo *sr10*KO	early_trophs	4	0.30 ± 0.02	24.45 ± 0.13	20.81 ± 0.11	22.37 ± 0.12
Pyo WT	early_trophs	4	0.31 ± 0.01	28.03 ± 0.68	15.69 ± 0.33	20.81 ± 0.25
