# Supplementary material for: Malaria parasites regulate intra-erythrocytic development duration via serpentine receptor 10 to coordinate with host rhythms
Source: Nat Commun. 2020 Jun 2;11:2763. doi: 10.1038/s41467-020-16593-y (PMC7265539; doi:10.1038/s41467-020-16593-y)
Supplement: Supplementary file 3 — Reporting Summary [file 41467_2020_16593_MOESM3_ESM.pdf]

nature research

Corresponding author(s): Arnab Pain, Sarah E. Reece, Richard Culleton

Last updated by author(s): Apr 22, 2020

Reporting Summary

Nature Research wishes to improve the reproducibility of the work that we publish. This form provides structure for consistency and transparency in reporting. For further information on Nature Research policies, see Authors & Referees and the Editorial Policy Checklist .

Statistics

For all statistical analyses, confirm that the following items are present in the figure legend, table legend, main text, or Methods section.

n/a	Confirmed	
☐	☒	The exact sample size (*n*) for each experimental group/condition, given as a discrete number and unit of measurement
☐	☒	A statement on whether measurements were taken from distinct samples or whether the same sample was measured repeatedly
☐	☒	The statistical test(s) used AND whether they are one- or two-sided<br>*Only common tests should be described solely by name; describe more complex techniques in the Methods section.*
☒	☐	A description of all covariates tested
☐	☒	A description of any assumptions or corrections, such as tests of normality and adjustment for multiple comparisons
☐	☒	A full description of the statistical parameters including central tendency (e.g. means) or other basic estimates (e.g. regression coefficient) AND variation (e.g. standard deviation) or associated estimates of uncertainty (e.g. confidence intervals)
☐	☒	For null hypothesis testing, the test statistic (e.g. *F*, *t*, *r*) with confidence intervals, effect sizes, degrees of freedom and *P* value noted<br>*Give P values as exact values whenever suitable.*
☒	☐	For Bayesian analysis, information on the choice of priors and Markov chain Monte Carlo settings
☒	☐	For hierarchical and complex designs, identification of the appropriate level for tests and full reporting of outcomes
☐	☒	Estimates of effect sizes (e.g. Cohen's *d*, Pearson's *r*), indicating how they were calculated

Our web collection on statistics for biologists contains articles on many of the points above.

Software and code

Policy information about availability of computer code

Data collection	RNASeq data were generated from Illumina HiSeq 4000 platform
Data analysis	FASTQC, TopHat2 (version 2.0.13), HTSeq- count, EdgeR, DeSeq2, JTK-Cycle, ARSER, DEXSeq program v 1.20.02 and R softwares were used for data analysis. All other details of the code used in the study are available from the corresponding author upon reasonable request.

For manuscripts utilizing custom algorithms or software that are central to the research but not yet described in published literature, software must be made available to editors/reviewers. We strongly encourage code deposition in a community repository (e.g. GitHub). See the Nature Research guidelines for submitting code & software for further information.

Data

Policy information about availability of data

All manuscripts must include a data availability statement. This statement should provide the following information, where applicable:

- Accession codes, unique identifiers, or web links for publicly available datasets
- A list of figures that have associated raw data
- A description of any restrictions on data availability

RNASeq data sets associated with this study have been submitted to the gene expression omnibus under accession numbers GSE132647, GSE144976 . See also Supplementary Data 1, 3 and 4. The source data underlying Figs. 1d, 1e, 1f, 3e, 3f, 3g, 3h, 4d, 4e, Supplementary Figs. 1d, 2c, 6c and 6d are provided as Source Data file.

Field-specific reporting

Please select the one below that is the best fit for your research. If you are not sure, read the appropriate sections before making your selection.

☒ Life sciences ☐ Behavioural & social sciences ☐ Ecological, evolutionary & environmental sciences

For a reference copy of the document with all sections, see nature.com/documents/nr-reporting-summary-flat.pdf

Life sciences study design

All studies must disclose on these points even when the disclosure is negative.

Sample size	Time series life-stage counts for sr10KO lines were all done with four replicates (CBA mice). Life-stage counting with 4-5 replicates have been done routinely in the laboratory and have previously yielded consistent results. 3 hour timepoints were chosen for taking smears and blood microsamples in order to minimize stress to the animals and allow for sufficient time for the mice to recuperate.
Data exclusions	We have excluded data for failed experiments and data with inadequate sequence read numbers that we thought will fail to provide us with reliable and biologically reproducible results.
Replication	We used 2-4 biological replicates depending on the type of experiments. All attempts of replication were successful.
Randomization	For both mismatch and sr10KO experiment, mice were randomly allocated to sample groups. Sample collection at each timepoint was randomized both at replicate and group levels.
Blinding	Investigators were blinded to group allocation during the data collection and analysis for microscopic slide reading for count of parasite stages. For both mismatch and sr10KO experiments investigators were blinded to group allocation during sample collection, sample processing, data collection and analysis

Reporting for specific materials, systems and methods

We require information from authors about some types of materials, experimental systems and methods used in many studies. Here, indicate whether each material, system or method listed is relevant to your study. If you are not sure if a list item applies to your research, read the appropriate section before selecting a response.

Materials & experimental systems

n/a	Involved in the study
☒	☐ Antibodies
☒	☐ Eukaryotic cell lines
☒	☐ Palaeontology
☐	☒ Animals and other organisms
☒	☐ Human research participants
☒	☐ Clinical data

Methods

n/a	Involved in the study
☒	☐ ChIP-seq
☒	☐ Flow cytometry
☒	☐ MRI-based neuroimaging

Animals and other organisms

Policy information about studies involving animals; ARRIVE guidelines recommended for reporting animal research

Laboratory animals	6-8 week old female MF1 mice were housed in open top cages at 21°C and 65% humidity. Light/dark cycle info is described extensively in the paper already (“One group (termed “matched”) were entrained to the same photoperiod as the donor mice, generating infections in which parasite and host rhythms were in the same phase. The other group (termed “mismatched”) was entrained to reverse light (Lights OFF 07:30 and Lights ON 19:30 GMT) resulting in infections in which the parasite is out of phase with the host by 12 hours.”)<br><br>Female CBA inbred mice (6-8 weeks old) housed at 23°C with 12hr light-dark cycle (lights-off: 19:00 h and lights-on: 07:00 h) and fed on a maintenance diet with 0.05% PABA-supplemented water.<br><br>Female ICR mice (6-8 weeks old), housed at 23°C with 12hr light-dark cycle (lights-off: 19:00 h and lights-on: 07:00 h) and fed on a maintenance diet with 0.05% PABA-supplemented water.
Wild animals	No wild animals used in this study
Field-collected samples	No field collected samples used in this study

Ethics oversight	All the procedures for the parasite mismatching study were performed in accordance with the UK Home office regulations (Animals Scientific Procedures ACT 1986: project license number 70/8546) and approved by the University of Edinburgh. All the procedures for the sr10ko study were performed in strict accordance with the Japanese Humane Treatment and Management of Animal Law (Law No. 105 dated October 1973 modified on 2 June, 2006), the Regulation on Animal Experimentation at Nagasaki University, Japan. The protocol was approved by Animal Experimentation Committee of Nagasaki University (permit: 12072610052). The animal experiments conducted in Edinburgh and Nagasaki were officially exempted from additional IBEC clearances in KAUST.

Note that full information on the approval of the study protocol must also be provided in the manuscript.
