# Supplementary material for: Malaria parasites regulate intra-erythrocytic development duration via serpentine receptor 10 to coordinate with host rhythms
Source: Nat Commun. 2020 Jun 2;11:2763. doi: 10.1038/s41467-020-16593-y (PMC7265539; doi:10.1038/s41467-020-16593-y)
Supplement: Supplementary file 4 — Description of Additional Supplementary Files [file 41467_2020_16593_MOESM4_ESM.pdf]

Description of Additional Supplementary Files

File Name: Supplementary Data 1
Description: Genes with daily expression detected in host rhythm matched and mismatched *P. chabaudi* parasites.

File Name: Supplementary Data 2
Description: Enriched gene ontology terms associated with host-cues responsive daily rhythmic genes.

File Name: Supplementary Data 3
Description: Genes with putative circadian expression detected in *P. falciparum* free running condition.

File Name: Supplementary Data 4
Description: Genes with daily rhythmic expression detected in *P. chabaudi chabaudi* AS wild-type and sr10KO parasites.

File Name: Supplementary Data 5
Description: Enriched gene ontology terms associated with SR10 linked daily rhythmic genes.

File Name: Supplementary Data 6
Description: Differentially regulated genes identified in sr10KO parasites compared to wild parasites in 4 matching time points.

File Name: Supplementary Data 7
Description: Genes for which differential alternative splicing events detected in two time points of sr10KO parasites compared to wild parasites.

File Name: Supplementary Data 8
Description: Sequence of primers used in this study.

File Name: Supplementary Data 9
Description: Zipped file containing raw Sanger sequencing data confirming the successful knockout of sr10 in *P. chabaudi* and *P. yoelii* sr10KO parasites.
