# Supplementary figures and images for: Malaria parasites regulate intra-erythrocytic development duration via serpentine receptor 10 to coordinate with host rhythms
Source: Nat Commun. 2020 Jun 2;11:2763. doi: 10.1038/s41467-020-16593-y (PMC7265539; doi:10.1038/s41467-020-16593-y)

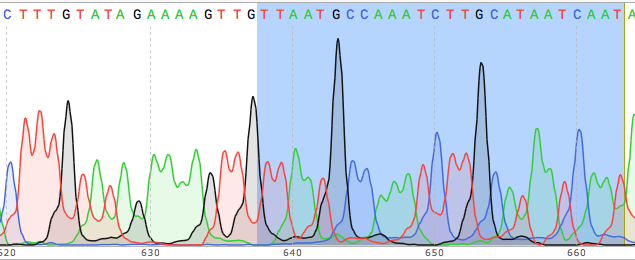

Supplement: Supplementary file 13 — Supplementary Data 9 [file 41467_2020_16593_MOESM13_ESM.zip › Supplementary Data 9/P. yoelii 3' integration.png]

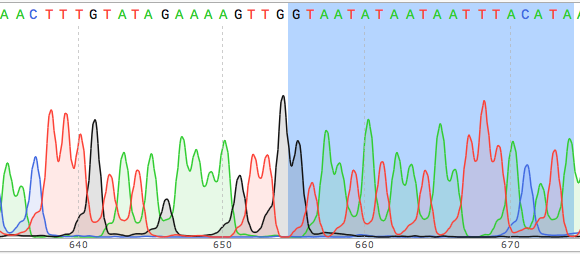

Supplement: Supplementary file 13 — Supplementary Data 9 [file 41467_2020_16593_MOESM13_ESM.zip › Supplementary Data 9/P. chabaudi 3' integration.png]

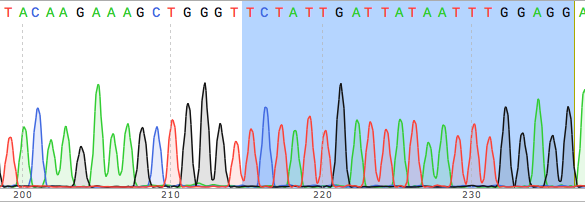

Supplement: Supplementary file 13 — Supplementary Data 9 [file 41467_2020_16593_MOESM13_ESM.zip › Supplementary Data 9/P. chabaudi 5' integration.png]

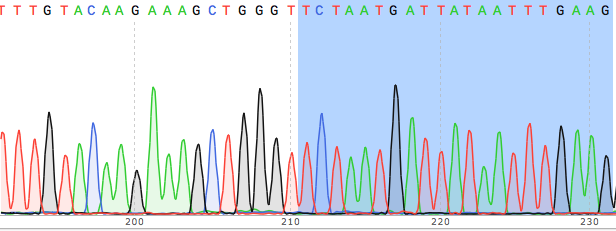

Supplement: Supplementary file 13 — Supplementary Data 9 [file 41467_2020_16593_MOESM13_ESM.zip › Supplementary Data 9/P. yoelii 5' integration.png]
